# Handheld multispectral photoacoustic imaging for assessing myocardial metabolism

**DOI:** 10.1117/1.JBO.30.10.105001

**Published:** 2025-10-07

**Authors:** Bokang Zhai, Yawen Shi, Handi Deng, Hongli Liu, Chenliang Xie, Naiyue Zhang, Wenyuan Yu, Dingce Sun, Yang Yu, Cheng Ma

**Affiliations:** a Beijing Anzhen Hospital, Capital Medical University, Department of Cardiac Surgery, Beijing, China; b Tsinghua University, Beijing National Research Center for Information Science and Technology, Department of Electronic Engineering, Beijing, China; c Tsinghua University, Institute for Precision Healthcare, Beijing, China; d IDG/McGovern Institute of Brain Research, Beijing, China

**Keywords:** photoacoustic imaging, myocardial metabolism, cardiac surgery, ischemic heart disease

## Abstract

**Significance:**

Myocardial oxygen metabolism is a key focus of cardiac surgery. It serves as important evidence for surgeons to evaluate surgical quality and surgical plans. However, current clinical methods lack the capability to directly monitor dynamic changes in myocardial metabolism during surgery. Photoacoustic imaging (PAI), a biomedical optical imaging modality, offers real-time assessment of blood oxygen saturation. By visualizing oxygen saturation levels in both blood and muscle tissue, PAI provides a means to infer myocardial metabolic status intraoperatively.

**Aim:**

We use PAI to observe the differences between infarcted myocardium and normal cardiac muscle and to explore the feasibility of using PAI to monitor myocardial metabolism levels during cardiac surgery.

**Approach:**

Ten rabbits were randomly divided into experimental and control groups. The animals in the experimental group underwent thoracotomy followed by left anterior descending coronary artery ligation, whereas those in the control group received thoracotomy only. PAI was performed both at the beginning and before the end of the surgical procedure. The PAI results were compared between the two groups to analyze the relationship between myocardial PAI signal changes and oxygen metabolism levels.

**Results:**

Following coronary ligation, the experimental group exhibited significant ST-segment elevation on electrocardiography, whereas no notable changes were observed in controls. PAI demonstrated: baseline myocardial oxygen saturation (${\mathrm{SmO}}_{2}$) ranged from 45% to 72% across all rabbits. Ligation-induced ischemia sharply reduced ${\mathrm{SmO}}_{2}$ to 1% to 19% in experimental animals. Control animals maintained stable ${\mathrm{SmO}}_{2}$ levels throughout the procedure. Histopathological examination confirmed extensive myocardial necrosis in the apical region of ligated rabbits, consistent with the observed functional and metabolic alterations.

**Conclusions:**

PAI can detect myocardial oxygen saturation in real-time during surgery and determine the occurrence of myocardial ischemia and changes in oxygen metabolism levels based on differences in oxygen saturation.

## 1 Introduction

Ischemic heart disease affects over 200 million people worldwide. Coronary heart disease (CHD) is one of the most severe forms of ischemic heart disease. It not only requires long-term drug therapy but also carries a high risk of sudden death due to acute myocardial infarction. It has already become a significant economic burden on society.^1^ CHD can lead to stenosis and occlusion of coronary arteries, resulting in acute angina and sudden death. Patients with severe CHD have usually experienced myocardial infarction. Their myocardial tissues can be classified into normal myocardium, hibernating myocardium, and infarcted myocardium-based differences in blood supply and metabolic levels. Normal myocardial tissues have adequate blood supply and normal metabolic levels. By contrast, hibernating myocardial tissues, due to the lack of adequate blood supply, remain in a state of low oxygen consumption and metabolic levels to sustain survival.^2^^,^^3^ Infarcted myocardial tissues experience necrosis and metabolic shutdown as a result of a prolonged absence of blood supply.^4^ The diagnosis and treatment of CHD are the key points of clinical work. Coronary artery bypass surgery (CABG) is one of the important treatment options for CHD. The purpose of the surgery is to achieve revascularization, which saves both normal and hibernating myocardial tissues and improves their metabolic levels to enhance their contract function.^5^^,^^6^ For regions that have already undergone complete infarction, abnormal blood flow perfusion may exacerbate cardiac damage. This is why surgeons need to use different methods to help with the surgical plan. Currently, doctors use coronary computed tomography (CT), coronary angiography (CAG), and positron emission tomography-computed tomography (Pet-CT) before CABG.^7^ During the surgery, we typically use electrocardiogram (ECG), flowmeters, and high-frequency ultrasound to estimate the coronary condition and graft blood flow.^8^^,^^9^ However, these methods still fail to locate the obstructed vessels and ischemic regions on the heart surface or directly evaluate whether myocardial perfusion and metabolism have improved before closing the chest. Therefore, we need a radiation-free and real-time imaging method to evaluate myocardial metabolic levels during the surgery.

Myocardial oxygen metabolism predominantly relies on oxygen supplied by coronary circulation via hemoglobin-bound transport.^10^^,^^11^ During ischemic conditions, myoglobin-mediated oxygen release in cardiomyocytes provides transient buffering, yet its reserves are exhausted within seconds.^12^^–^^14^ Consequently, the regional concentration of oxygenated hemoglobin serves as an indicator for myocardial oxygen metabolism.

Photoacoustic imaging (PAI) is a biomedical optical imaging method capable of assessing blood oxygen saturation.^15^^–^^17^ Hemoglobin is closely related to the level of myocardial oxygen metabolism,^18^ which generally exists in two conformations: deoxygenated hemoglobin (Hb) and oxygenated hemoglobin (${\mathrm{HbO}}_{2}$). During myocardial ischemia, small blood vessels in the myocardium are in a state of low oxygen partial pressure, leading to the deoxygenation of large amounts of ${\mathrm{HbO}}_{2}$ into Hb.^19^ Therefore, the level of oxygen metabolism in muscle tissue can be evaluated based on the relative concentration of ${\mathrm{HbO}}_{2}$.

Previous studies have demonstrated that PAI can perform noninvasive myocardial imaging in mouse.^20^^,^^21^ However, these studies only applied the PAI over the mouse chest, and cardiac contractions in the mice are much weaker than those in large animals. Larger body size leads to greater myocardial contraction amplitude and stronger noise signals, consequently requiring modifications in signal acquisition methods. Some studies have used fluorescently labeled red blood cells as tracers, which were injected into CHD mice and successfully visualized the sites of vascular blockage.^22^ However, this method mainly focuses on blood perfusion and cannot reflect oxygen metabolism. Some studies on larger animal models have focused on systemic hypoxia and peripheral vascular blood oxygen saturation.^23^ Our study introduces a localized ischemia-reperfusion injury model that more closely mimics the clinical scenario of acute myocardial infarction and revascularization. This allows us to investigate spatially heterogeneous metabolic responses within the risk zone. Whether PAI can effectively image myocardial tissues in larger animals remains unexplored in further studies. Furthermore, due to the size limitations of mice, the established myocardial infarction models often exhibit large-scale myocardial infarction. In clinical practice, CAG can adequately assess the ischemic area in patients with large-scale myocardial infarction due to their pronounced symptoms. We anticipate that PAI may enable the evaluation of clinically subtle, more localized or occult coronary vascular diseases with insignificant symptoms. In this study, we induced a clinically representative acute myocardial infarction model in the left ventricular anterior wall and apex by ligating the midsegment of the rabbit’s left anterior descending coronary artery (LAD). In addition, in this study, we performed direct imaging of the exposed rabbit heart using a handheld PAI probe to simulate an authentic surgical scenario. The major challenges of this work include designing and implementing a small PAI probe that can be adapted intraoperatively and fit the surface of the heart, and performing multi-spectral imaging and estimating the oxygenation level in beating hearts. Our goal was to investigate the feasibility of using PAI for intraoperative monitoring of myocardial metabolic levels during cardiac surgery.

## 2 Materials and Methods

### 2.1 Myocardial Metabolic Assessment Based on PAI

In this study, we established a rabbit model of acute myocardial infarction and employed multiple clinical modalities (electrocardiography, echocardiography, histopathology) combined with PAI to quantitatively assess myocardial metabolic levels and identify ischemic regions.

### 2.2 Imaging System Design

**Fig. 1:**
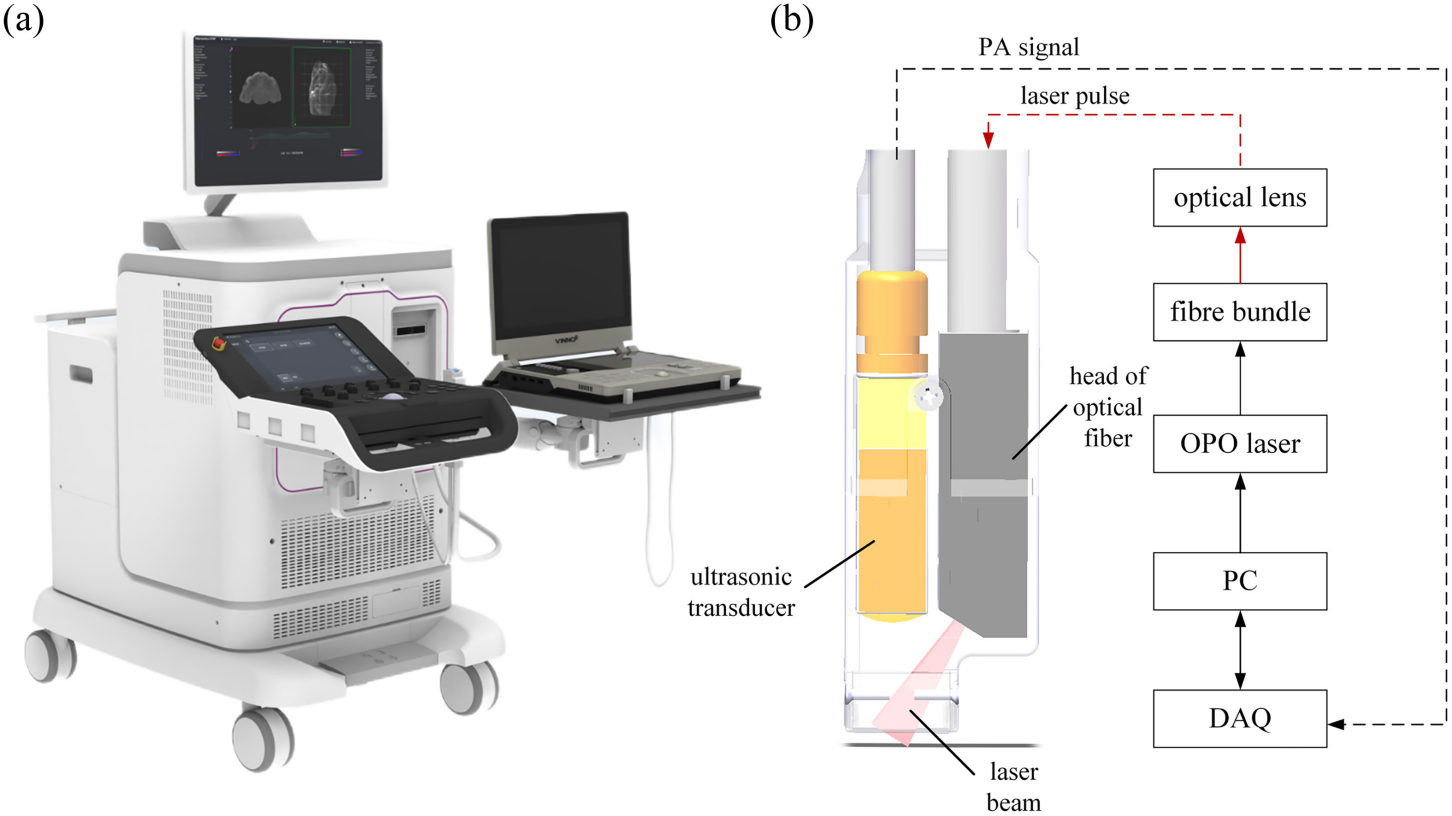
(a) Multi-wavelength photoacoustic imaging system used in this study. (b) A small handheld photoacoustic probe consisting of an ultrasonic transducer and an optical fiber bundle. PA, photoacoustic; OPO, optical parameter oscillator; PC, personal computer; DAQ, data acquisition system.

This study utilizes a self-developed photoacoustic-ultrasound dual-modality imaging system (Fig. 1). The system comprises a custom-designed multi-wavelength PA module and a commercial small animal ultrasound imaging module. The PAI module can perform both structural PA imaging and multi-wavelength functional PA imaging, whereas the ultrasound imaging module supports various ultrasound imaging modes (B-mode, C-mode, and M-mode). Considering the different requirements for the central frequency of probes in PA and ultrasound imaging for cardiac imaging, we use probes with different frequency bands for signal acquisition during PA and ultrasound imaging. The primary components of the multi-wavelength PA imaging system include an optical parametric oscillator (OPO), a PAI probe, a data acquisition system (DAQ), and a computer. An OPO (Lasersound-10, Tsingpai Co., Ltd.) was employed, operating at a pulsed wavelength range of 680–980 nm with a pulse width of 5 to 7 ns, a repetition rate of 10 Hz, and a maximum single-pulse energy of 100 mJ. The system integrates a real-time energy monitoring module (ES120 energy sensor, Thorlabs, Inc.) and a wavelength calibration module (Aurora4000 spectrometer, resolution: 0.1 nm, Changchun New Industries Optoelectronics Tech. Co.), ensuring stable excitation light parameters.

The PAI probe, as shown in Fig. 1, consists of a 64-element ultrasonic transducer (center frequency: 5 MHz, bandwidth: 60%, AuSen Technology), a multimode fiber bundle (output area: 2×18  mm, NA = 0.22, Ruihe Aerospace) and a custom-designed coupling pad. Randomization of the fiber bundle results in a spatially uniform rectangular illumination pattern (20  mm×10  mm) with energy density maintained at $10\;\mathrm{mJ}/cm^{2}$, in compliance with the American National Standards Institute (ANSI) laser safety standards. The light inlet is protected by a window lens, and the light outlet adopts oblique light emission to allow intersection between acoustic sectioning and illumination, which helps improve the imaging quality. The probe surface is coated with a polydimethylsiloxane (PDMS) elastomeric membrane (thickness: 0.1 mm, acoustic impedance: 1.5 MRayl), whereas the interior is filled with purified water for acoustic coupling, ensuring compatibility with dynamic cardiac surface deformation.

#### 2.2.1 Data acquisition module

**Fig. 2:**
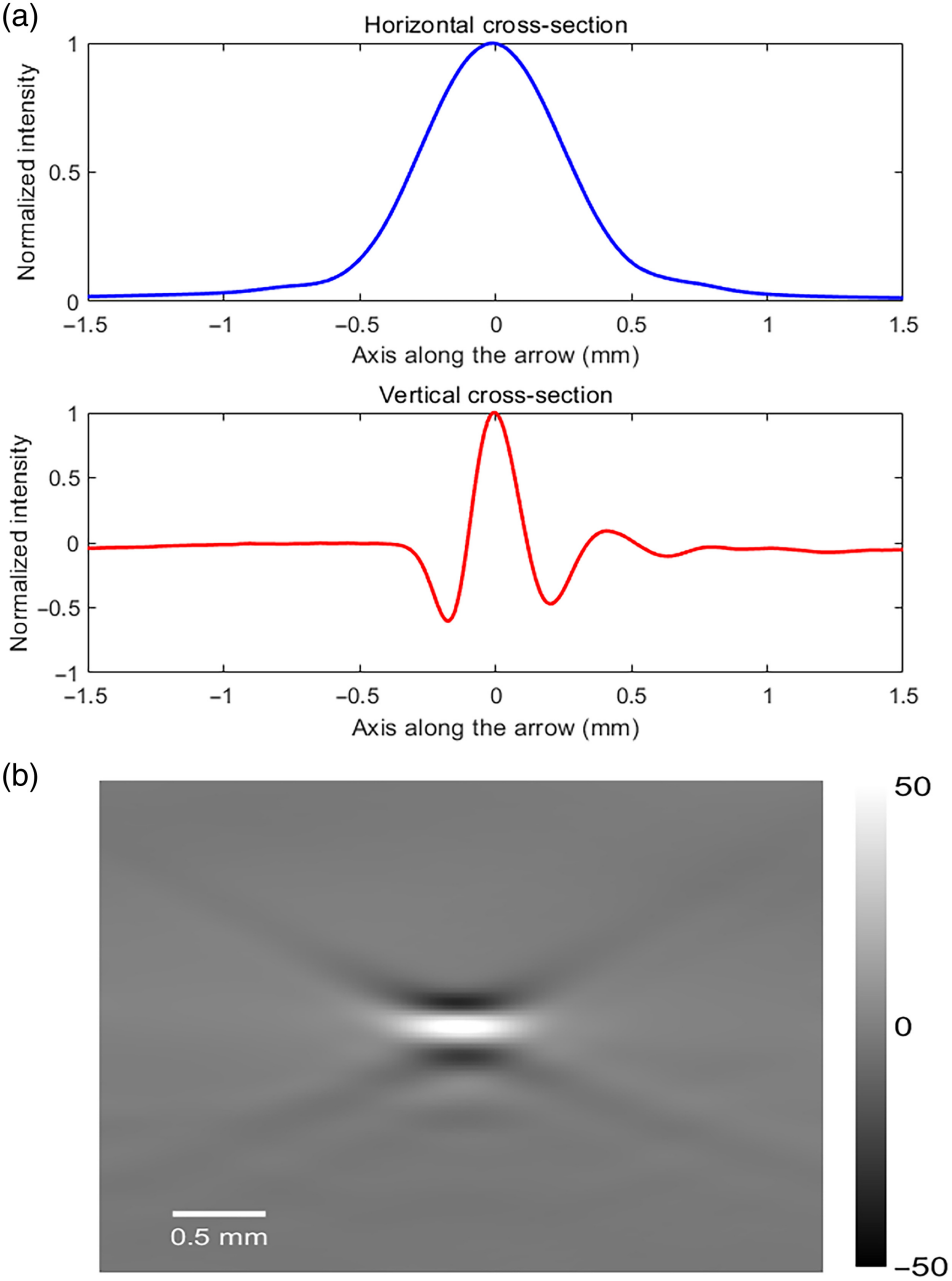
(a) Normalized one-dimensional photoacoustic intensity profiles along the horizontal (blue) and vertical (red) directions extracted from the center of the wire cross-section. (b) Photoacoustic cross-sectional image of a 10-μm diameter tungsten wire acquired with a 5 MHz linear array transducer.

The system integrates a 64-channel data acquisition card (Marsonics DAQ64, Tsingpai, China) operating at 40  MS/s sampling rate with 0.1 to 20 MHz analog bandwidth and 80 dB dynamic range. An optimized electromagnetic shielding architecture ensures robust performance, delivering 35 dB SNR as validated through standardized phantom measurements. In addition, the DAQ can receive data from the energy meter to monitor the light intensity of each excitation pulse, thereby correcting light energy fluctuations and improving spectral unmixing accuracy. The data collected by the DAQ is uploaded to a computer and processed and displayed using the image reconstruction and spectral unmixing algorithms based on Compute Unified Device Architecture (CUDA). Data collected by the spectrometer are also synchronized and uploaded to the computer for monitoring the optical wavelength. To quantify the spatial resolution capability of this PAI system, a tungsten wire with a diameter of 10  μm, aligned perpendicular to the imaging plane, was imaged as a point-source target. The system resolution was determined by measuring the zero-crossing width of the main lobe of the photoacoustic signal and subtracting the wire’s physical diameter.^24^ The measured lateral and axial resolutions were 1220 and 220  μm, respectively. Ultrasound images are acquired using the small animal ultrasound imaging module (VINNO 6 LAB, FeEno Medical Technology Co., Ltd., Suzhou, China) equipped with a probe with a central frequency of 17.5 MHz and a bandwidth of 65%. The device also has the capability of ECG acquisition. The acquired images and data are transmitted to the computer via High Definition Multimedia Interface (HDMI) and RJ-45 interfaces, facilitating the processing and fusion of multi-modality data (Fig. 2).

### 2.3 Animal Preparation

A total of 10 New Zealand white rabbits (weight, 2.5 to 3 kg; age, 5 to 6 months) were used in this study. All studies were approved by the Beijing Anzhen Hospital Medical Ethics Committee (AZ2025LA020).

Rabbits were randomly divided into experimental group and control group. Using 5  mL/kg of Zoletil-50 (intramuscular injection) and 0.1  mg/kg of Meloxicam (intravenous injection) for anesthesia and analgesia in rabbits. Then, the rabbits were fixed on the operating table, shaved, and connected to an ECG. The neck skin was cut open to expose the trachea, and tracheal intubation was performed. The ventilator was used for assisted ventilation with a tidal volume of 40 mL, a respiratory rate of 30 times/min, and a respiratory ratio of 1:1.5. The ventilator was supplied with ambient air only, without supplemental oxygen or other gas mixtures. The sternum was cut open along the midline of the sternum, the pericardium was opened, and the heart was exposed.

### 2.4 Data Acquisition

The chest was entered through a standard median sternotomy, with meticulous pleural preservation. The pericardium was incised to expose the heart, followed by cardiac elevation with saline gauze to optimize exposure. We observed the motion of the heart and recorded the ECGs. Transthoracic echocardiography was performed using the VINNO 6 LAB ultrasound system. To obtain the PA image of the myocardium, we used the handheld PAI probe and applied ultrasound coupling agent on its surface to ensure good contact with the heart’s surface. The muscle oxygen saturation (${\mathrm{SmO}}_{2}$) represents the total amount of oxygen carried by hemoglobin in muscle tissue. Based on the near-infrared light absorption characteristics of Hb and ${\mathrm{HbO}}_{2}$, there is a significant difference in absorption between the two at 760 and 820 nm. Therefore, we primarily focus on the signals at these two wavelengths. Multi-wavelength excitation necessitating prolonged acquisition for adequate signal noise ratio (SNR). Due to the limited size of the imaging probe, prolonged scanning may compress the heart and impair myocardial function. With dual-wavelength imaging at a pulse repetition rate of 10 Hz, the system requires only 0.2 s to complete one acquisition cycle. This better accommodates the pulsatile nature of the heart, ensuring that most data acquisitions occur during non-systolic intervals, thereby improving the accuracy of the results. We attached the probe closely to the heart’s surface, set the acquisition frequency to 10 Hz, and alternately collected myocardial tomographic PA images at the two wavelengths of 760 and 820 nm after the laser energy was stabilized. The collection lasted for 30 s. We continuously collected 150 sets of data, totaling 300 frames.

Considering the potential systematic errors introduced by the unevenness of laser output energy and the difference in transmission efficiency of optical fibers for light energy at different wavelengths, we conducted real-time monitoring of laser energy during the signal acquisition process. In addition, we collected signals from a thin tube of copper sulfate having a known absorption spectrum, which served as a reference standard for calibrating spectral measurement errors.

After image acquisition, the rabbits in the experimental group underwent ligation of the mid-segment of the left anterior descending coronary artery located in the interventricular septum. After ligation, ECG changes were observed and recorded. The chest incision was covered with gauze, and 3 h later, the ejection fraction of the rabbits was re-measured using an ultrasound scanner, and the epicardial signals were re-detected using a PA probe.

Compared with the experimental group, the control group animals underwent thoracotomy without coronary artery ligation, but received PAI immediately after the thoracotomy. Three hours after thoracotomy, PA signals were collected again. After signal acquisition, myocardial specimens were collected, and the excised tissues were stained with hematoxylin and eosin (H&E). Microscope images of the stained tissues were captured at magnifications of 200× and 400× using an optical microscope.

### 2.5 Signal Reconstruction

In this study, the delay and sum (DAS) algorithm was utilized to reconstruct the ultrasonic signals,^25^ calculating the sum of all array signals corresponding to each pixel in the image, thereby obtaining the intensity information of the entire myocardial imaging area.

To reduce the calculation errors caused by the heart beat and improve the SNR, we made a series of efforts. Photoacoustic signals are susceptible to environmental electromagnetic interference (EMI) and intrinsic probe noise. In addition to conventional electromagnetic shielding, our specially designed probe structure can reduce acoustic impedance mismatch. The probe surface is coated with a PDMS elastomeric membrane, whereas the interior is filled with purified water for acoustic coupling, ensuring compatibility with dynamic cardiac surface deformation. When collecting signals, apply ultrasound coupling gel to the probe surface to eliminate acoustic impedance mismatch caused by air gaps. We set the acquisition frequency to 10 Hz and alternately collected myocardial tomographic PA images at the two wavelengths of 760 and 820 nm. High-frequency data acquisition of the heart was performed to observe the cardiac motion cycle after reconstruction. Data from specific periods were screened and time-averaged to obtain more stable signals, thereby reducing the impact of instantaneous fluctuations caused by heartbeat on the results. The experimental rabbits underwent endotracheal intubation to control their respiratory rate and tidal volume, ensuring that stable images could be obtained at specific respiratory phases.^26^

At the wavelength of 760 nm, 50 pixels within the region of the strongest myocardial PA signal were selected as the region of interest (ROI), and their average value was calculated. The same process was applied to the pixels in the same area at the wavelength of 820 nm. The myocardial PA spectrum was calibrated using the intensity difference between the measured and theoretical PA signals from the copper sulfate solution.

To obtain the true and accurate spectrum of an object, we use a known absorber, copper sulfate solution, to calibrate the photoacoustic spectrum. The copper sulfate solution is placed in a PTFE tube for measurement. Copper sulfate is stable at room temperature, does not evaporate or decompose easily, and its absorbance changes very little over long-term storage, making it suitable as a long-term calibration standard. Before each experiment, we are able to calibrate the system using this method. Assuming the absorption spectrum of the copper sulfate solution is $c_{0}$ (known), and the measured photoacoustic spectrum after data collection is $c_{0t}$, the system’s transmission spectrum can be expressed as

$$T=\frac{c_{0t}}{c_{0}}.$$ (1)

Then, for the test object, its actual absorption spectrum can be expressed as

$$c=\frac{c_{t}\cdot c_{0}}{c_{0t}}.$$ (2)

The difference in absorption characteristics between Hb and ${\mathrm{HbO}}_{2}$ at different wavelengths can be utilized to calculate oxygen saturation. Subsequently, a linear unmixing algorithm was employed to resolve the ratio between the oxygenated and the total amount of hemoglobin, enabling the calculation of myocardial oxygen saturation.^27^

Assuming P(λ) presents the local photoacoustic pressure at wavelength λ, $\alpha_{\text{oxy-Hb}}(\lambda )$, $\alpha_{\text{deoxy-Hb}}(\lambda )$ denote the molar absorption coefficients of oxy-hemoglobin and deoxy-hemoglobin, respectively, whereas $C_{\text{oxy-Hb}}$ and $C_{\text{deoxy-Hb}}$ are their respective molar concentrations. Inside the illuminated muscle, the following equation can be obtained:

$$P(\lambda )=q_{0}\cdot (C_{\text{oxy-Hb}}\cdot \alpha_{\text{oxy-Hb}}(\lambda )+C_{\text{deoxy-Hb}}\cdot \alpha_{\mathrm{deoxy}-\mathrm{Hb}}(\lambda )),$$

where $q_{0}$ is a conversion factor for unit consistency, which is the product of a series of factors

$$q_{0}=\Gamma \cdot \eta_{\mathrm{th}}\cdot \phi (\mu_{a},\mu_{s}),$$

where Γ is the Grünessen coefficient and $\eta_{\mathrm{th}}$ denotes the efficiency of light-to-heat conversion. Both parameters are typically treated as constants under uniform tissue conditions. $\phi (\mu_{a},\mu_{s})$ represents the actual optical fluence acting on the biological tissue, which is influenced by the global distribution of the absorption coefficient ($\mu_{a}$) and the scattering coefficient ($\mu_{s}$) within the tissue. Variations in the fluence spectra directly affect the photoacoustic spectra. In this study, the imaging target is myocardial tissue, with the ROI located in the superficial layer of the biological tissue. The photoacoustic signal strength in this region is influenced by light fluence to a much lesser extent compared to deeper tissues. Therefore, in this paper, it is assumed that ϕ is also a constant at a superficial depth of 3 mm. Under relatively ideal conditions, the model can be simplified by neglecting the factors contained in $q_{0}$ and assuming it to be a constant.

Because the molar absorption coefficients are known, solving a set of linear equations at different wavelengths allows us to calculate the saturation of oxygen in the muscle as defined in the following equation:

$$[\begin{array}{c} C_{\text{oxy-Hb}}\\ C_{\text{deoxy-Hb}} \end{array}]=\frac{1}{\kappa }{\left[\begin{array}{cc} \alpha_{\text{oxy-Hb}}(\lambda_{1}) & \alpha_{\text{deoxy-Hb}}(\lambda_{1})\\ \vdots & \vdots \\ \alpha_{\text{oxy-Hb}}(\lambda_{N}) & \alpha_{\text{deoxy-Hb}}(\lambda_{N}) \end{array}\right]}^{-1}[\begin{array}{c} P(\lambda_{1})\\ \vdots \\ P(\lambda_{N}) \end{array}],$$

where $P(\lambda_{i})$ presents the local photoacoustic pressure at wavelength λ and κ is a conversion factor for unit consistency. Muscle oxygen saturation (${\mathrm{SmO}}_{2}$) is defined as the ratio of oxygenated hemoglobin to total hemoglobin in muscle tissue

$${\mathrm{SmO}}_{2}=\frac{C_{\text{oxy-Hb}}}{C_{\text{deoxy-Hb}}+C_{\text{oxy-Hb}}}.$$

## 3 Results

### 3.1 Myocardial Function in PA and Ultrasound

**Fig. 3:**
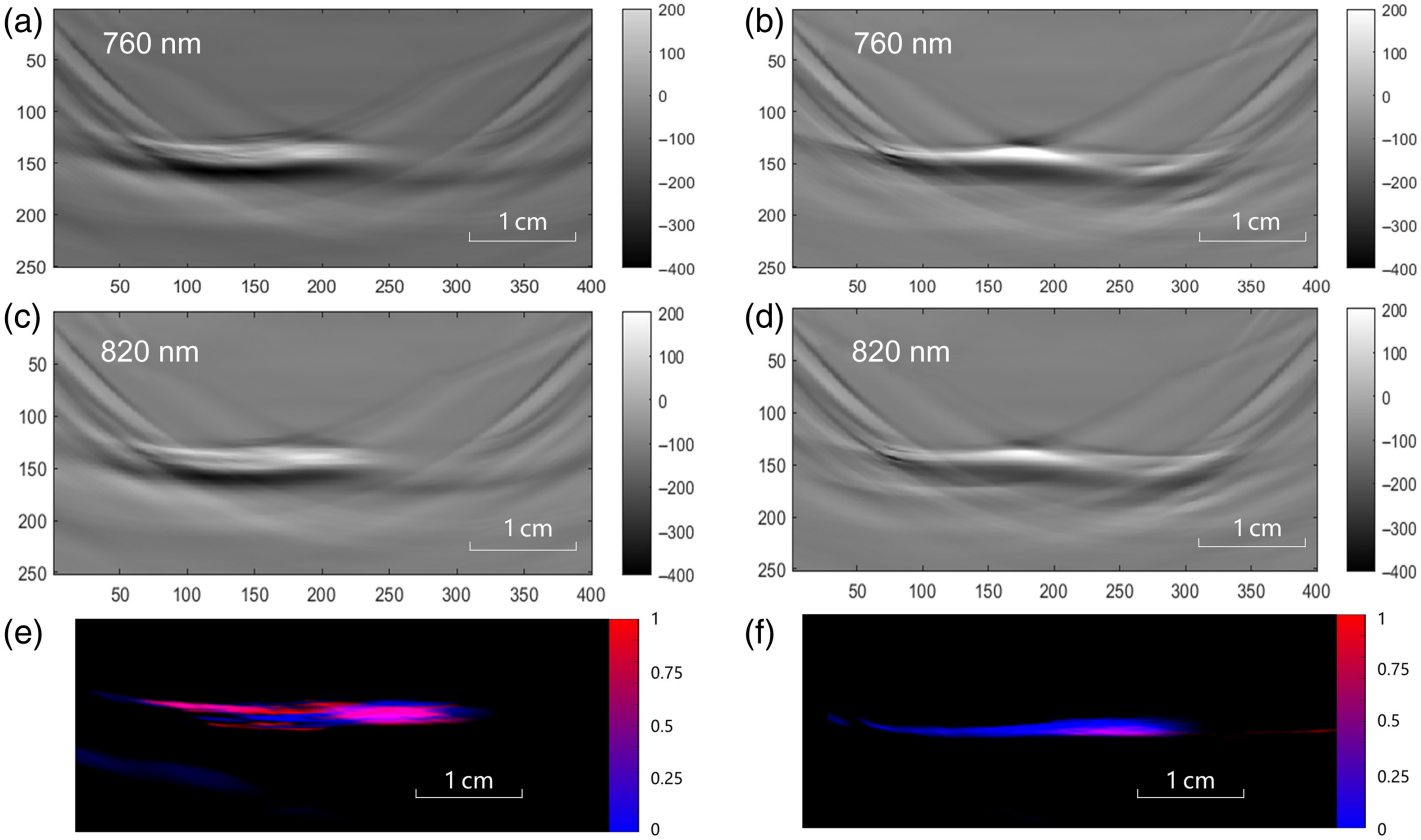
Photoacoustic images and reconstructed pseudo-color images: (a) pre-ligation photoacoustic images of the experimental group animals at 760 nm. (b) Post-ligation photoacoustic images of the experimental group animals at 760 nm. (c) Pre-ligation photoacoustic images of the experimental group animals at 820 nm. (d) Post-ligation photoacoustic images of the experimental group animals at 820 nm. (e) Pre-ligation pseudo-color images of the experimental animals. (f) Post-ligation pseudo-color images of the experimental animals.

In this study, no animals died during the entire experiment. Figure 3 displays the acquired photoacoustic images and the oxygen saturation pseudo-color images of PAI. Panels a, b, c, and d present the photoacoustic images of experimental animals at 760 and 820 nm wavelengths, both pre- and post-ligation. The pseudo-color images in panels e and f were obtained by calculating myocardial tissue oxygen saturation within the photoacoustic images.

**Fig. 4:**
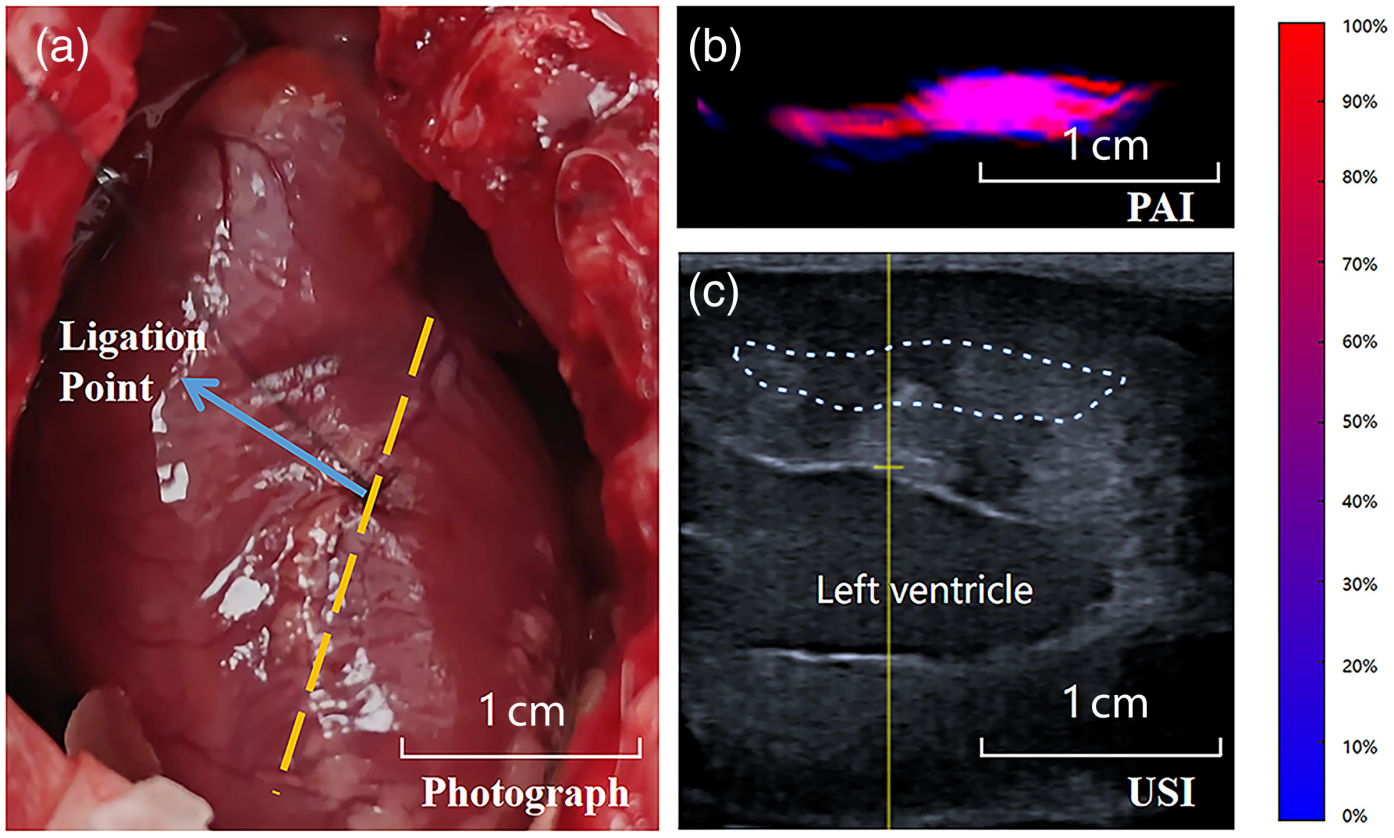
Three different methods for observing the myocardium: (a) Picture of the exposed rabbit heart. The yellow dashed line marks the cross-section for acquiring photoacoustic images and echocardiograms, and the blue line indicates the location of the ligation point. (b) Reconstructed pseudocolor map of myocardial oxygen saturation from photoacoustic imaging data. (c) Echocardiogram: the area within the white dashed lines represents the acquisition range of the pseudocolor map.

**Fig. 5:**
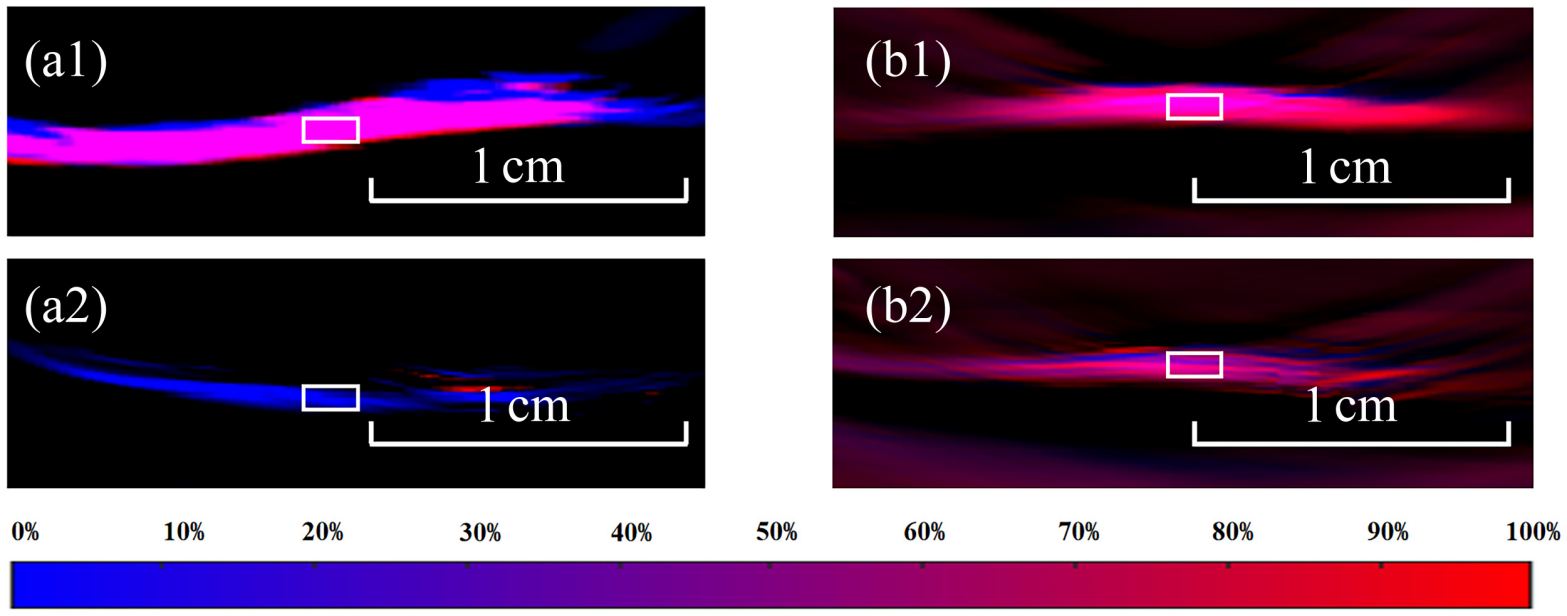
Pseudocolor map of myocardial oxygen saturation. (a1) and (a2) display the pseudocolor maps of myocardial oxygen saturation at the beginning and end of the experiment for the experimental group, respectively, whereas (b1) and (b2) show the corresponding maps for the control group. The white box delineates the ROI (region of interest) for oxygen saturation calculation.

In this study, PAI provided information on the ratio of ${\mathrm{HbO}}_{2}$ in the myocardium. Figure 4 shows heart images acquired using different methods. The cross-sections where we acquired the PA and echocardiographic images were marked in the photo shown in Fig. 4(a). We acquired the PA signal along the yellow dashed line in Fig. 4(a) and then calculated the ratio of ${\mathrm{HbO}}_{2}$ to the total hemoglobin to create the quantitative PA image as shown in Fig. 4(b). Figure 4(c) represents the corresponding ultrasound image at the same cross-section. After acquiring images, the experimental rabbits underwent ligation as shown in the figure. The ratio between the oxygenated and the total hemoglobin was calculated from the PA signals. Figure 5 displays myocardial oxygen saturation in both experimental and control groups at baseline and study termination. Quantitative analysis of the ROI regions revealed a significant decrease in oxygen saturation 3 h post-ligation in the experimental group, whereas no significant changes were observed in control animals.

**Fig. 6:**
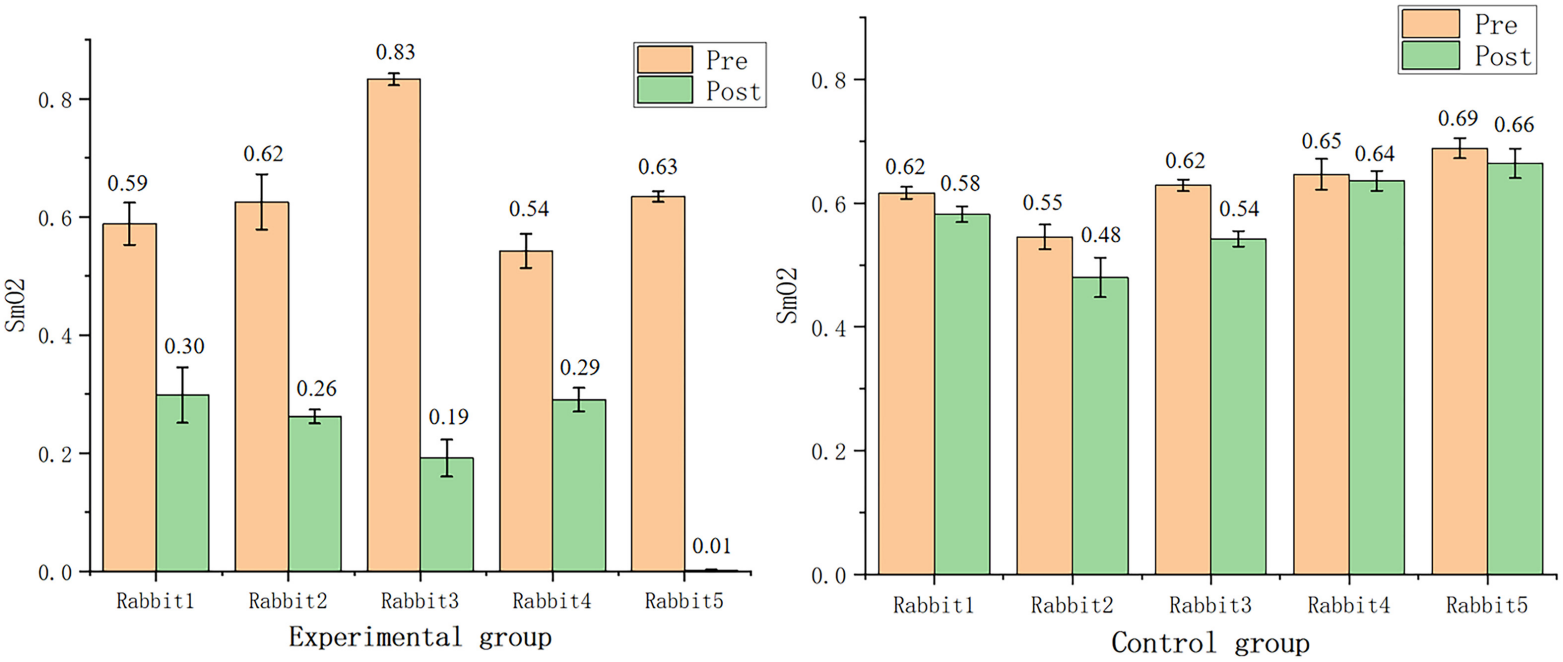
Bar plots of estimated ${\mathrm{SmO}}_{2}$ values. The left image shows the results of the experimental group, the right image shows the results of the control group.

After the PA images were reconstructed, the values of were calculated, and the comparison charts were drawn to highlight the difference before and after ligation, as shown in Fig. 6. It is apparent that the ${\mathrm{SmO}}_{2}$ values under normal conditions were between 45% and 72%, and dropped dramatically to 1% to 19% after ligation. By contrast, in the control group, the ${\mathrm{SmO}}_{2}$ values did not change significantly.

After statistical verification, the ${\mathrm{SmO}}_{2}$ values measured at the beginning in the experimental group and the control group conformed to the normal distribution. The comparison of the variances of the ${\mathrm{SmO}}_{2}$ values between the experimental group and the control group at the start was unequal (P=0.085<0.1). The results of the t-test without assuming equal variances indicated that the difference in the ${\mathrm{SmO}}_{2}$ values between the experimental group and the control group at the beginning of the experiment was not statistically significant (P=0.755>0.05), which meant that the rabbits in the experimental group and the control group were in the same state at the beginning of the surgery. The differences in the ${\mathrm{SmO}}_{2}$ values were calculated separately for the experimental and control groups, and both conformed to the normal distribution. The Levene’s test suggested that the variances were equal (P=0.11>0.1). The results of the t-test indicated that the difference in the proportion changes between the experimental group and the control group was statistically significant (P=0.001<0.05); therefore, the ligation operation would lead to a decrease in the myocardial oxygenated protein content measured by PAI in rabbits.

Echocardiography reflected the differences in myocardial function and motion before and after ligation in the experimental group. In the experimental group, all rabbits showed decreased ejection fraction and reduced motion of the anterior left ventricular wall after ligation. In addition, in the experimental group rabbits, ST-segment elevation was observed on the ECG after coronary ligation, indicating ischemia from the epicardium to the middle layer of the myocardium. Therefore, the changes in PA images originated from myocardial ischemia caused by the ligation procedure, and the regions with weakened PA signals exhibited decreased myocardial contractility.

### 3.2 Myocardial Pathology

**Fig. 7:**
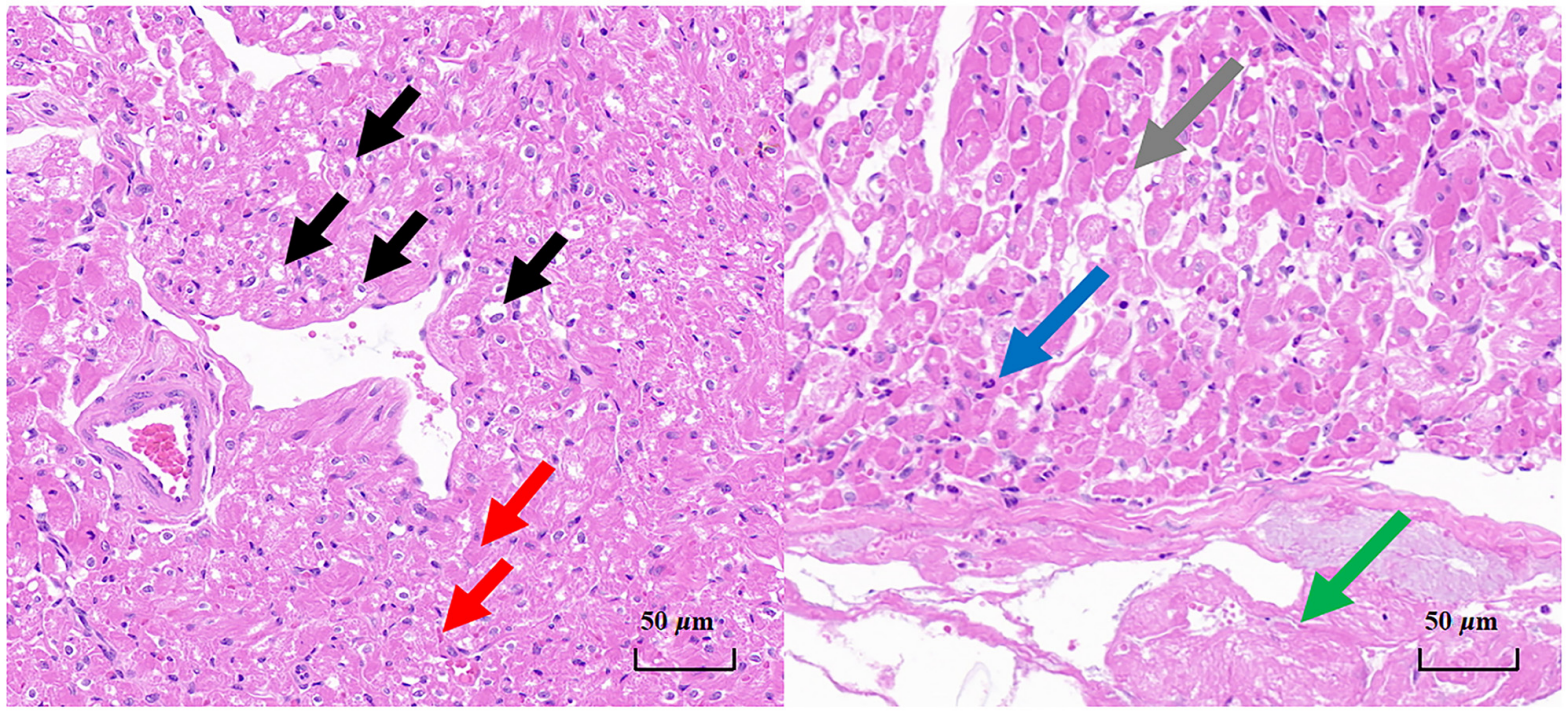
Histopathology images of specimens taken from the myocardial ischemia area. Black arrows, myocardial cell degeneration; red arrows, myocardial cell necrosis; gray arrow, myocardial cell atrophy; blue arrow, granulocyte infiltration; green arrow, fibrin.

Figure 7 shows the pathological images of the specimens taken along the coronary artery in the experimental group. These specimens were examined under a microscope after H&E staining and were digitally recorded. The cardiomyocytes near the proximal end of the heart were basically normal, whereas a large number of platelets were found to accumulate in the intercellular space at the distal end of the heart. Numerous cardiomyocytes were found to be degenerated (black arrows), with loose and lightly stained cytoplasm, partial cytoplasmic vacuolation, and necrosis (red arrows) characterized by nuclear dissolution, cytoplasmic disintegration, and accompanied by minimal granulocyte infiltration (blue arrows). A smaller number of cardiomyocytes showed atrophy (gray arrows), with reduced cell volume. Insoluble fibrin was observed attached to the edge of focal tissue (green arrows). No significant abnormalities were found in interstitial blood vessels. The myocardial pathological images revealed extensive cell degeneration and necrosis within the ischemic area of the myocardium. Cells under severe ischemia and hypoxia exhibited cell swelling, membrane disruption, loss of normal morphology, and inability to perform normal metabolic functions. Therefore, the pathological results suggest a significant decline in metabolic function within the ischemic area after ligation.

PAI demonstrated that the myocardial PA signals in the experimental and control groups were basically at the same level under open-chest conditions; however, the myocardial PA signals in the experimental group significantly decreased after ligation, whereas those in the control group remained unchanged over time. This result indicates that changes in PA signals reflect the impact of ligation on the myocardium. Clinical assessment results showed that there are varying degrees of myocardial ischemia and metabolic abnormalities observed in the experimental rabbits after ligation. The elevation of ECG ST-segment indicates ischemia in the epicardium or the entire myocardium: sustained ischemia and hypoxia lead to dysfunction of proton pumps on the cell membrane, with potassium ions moving from inside to outside the cell, resulting in decreased resting potential and significantly shortened action potential duration, which creates a potential difference with the endocardial cardiomyocytes, leading to ST-segment elevation.^28^^,^^29^ Therefore, combining the ECG and PAI results, it can be inferred that myocardial ischemia leads to changes in PAI signals. Echocardiography showed decreased left ventricular ejection fraction, indicating poor myocardial motion and reduced motion amplitude^30^^,^^31^: myocardial contraction requires adenosine triphosphate (ATP) energy to trigger myofilament sliding, and reduced contractility indicates insufficient energy for myofilament sliding, which means decreased cellular metabolic levels; myocardial pathology revealed that large population of cells near the apical side exhibiting swelling, membrane disruption, and degeneration, with a small portion of cells showing nuclear dissolution and necrosis. Sustained ischemia and hypoxia in these cells led to enhanced anaerobic glycolysis, calcium overload, and other phenomena, with some metabolites accumulating inside and outside the cells, affecting the normal function of organelles. Abnormal ion levels activated phospholipases, leading to the dissolution and destruction of cell organelles containing phospholipids such as mitochondria and cell membranes, further exacerbating cellular metabolic abnormalities.^32^^,^^33^ These cells lack normal metabolic functions. Combining echocardiography, pathological findings, and the PAI results, it can be concluded that the decreased myocardial metabolism after myocardial ischemia is the fundamental reason for the decreased ${\mathrm{SmO}}_{2}$ values, and this kind of imaging mode has the ability to assess myocardial metabolic levels.

Although ECG and echocardiogram are used to observe changes in myocardial function before and after myocardial infarction in the study, they cannot reflect subtle changes in myocardial metabolic levels during actual surgical procedures. In addition, assessing myocardial metabolism through the collection of pathological specimens increases the patient’s burden and surgical trauma, making it impractical to be applied intraoperatively to evaluate metabolic changes during surgery. Similarly, PET-CT used to assess myocardial metabolism is difficult to apply during surgery. Other methods, such as TTFM and CAG, only have the ability to evaluate perfusion levels. In contrast, PAI can directly reflect real-time changes in tissue metabolic levels without additional surgical trauma, making it a complementary diagnostic modality to other examinations.

## 4 Discussion and Conclusion

In China, the total number of patients with CHD has exceeded 10 million, and international heart society data indicates that the number of patients with ischemic heart disease worldwide has surpassed 200 million, with projections of reaching over 450 million by 2045. For patients with severe CHD, CABG is a primary treatment option. Currently, China performs over 60,000 CABG surgeries annually, and this number continues to rise. CABG aims to restore normal metabolic and contractile functions of ischemic myocardium through revascularization.^5^^,^^34^ Preoperative metabolic evaluation assists surgeons in identifying optimal revascularization targets. Postoperative metabolic assessment serves as a critical indicator of surgical efficacy. However, current clinical modalities lack real-time intraoperative metabolic monitoring capability. PAI emerges as a promising solution to this clinical gap. In this study, we established a myocardial infarction model and performed comparative evaluations using conventional clinical assessments alongside PAI. Quantitative analysis demonstrated PAI’s ability to reliably differentiate normal from infarcted myocardium through ${\mathrm{SmO}}_{2}$ quantification (p<0.01). This study demonstrates the clinical utility of a handheld multispectral PAI system for intraoperative real-time monitoring of myocardial metabolic status.

We performed PAI of the superficial myocardial muscle using established spectral parameters. In this study, it was found that the proportion of ${\mathrm{HbO}}_{2}$ measured by PAI at the beginning of the surgery were 45% to 72%. These values are lower than arterial blood levels and peripheral venous blood levels. This difference arises because the ROI targets the myocardium rather than specific vascular structures (arteries or veins). The myocardium comprises arterioles, venules, and capillaries. The venous blood of myocardium drains into the coronary sinus, which normally exhibits a venous oxygen saturation of ∼30% under physiological conditions.^35^^,^^36^

After coronary artery ligation for 3 h, the PA signals of rabbits decreased at all wavelengths. This is because after ligation, not only was there hypoxia caused by oxygen consumption but also blood reduction in the capillary bed, indicating the coexistence of ischemia and hypoxia. Ischemia leads to the reduction of hemoglobin in the area, resulting in a decrease in signals at all wavelengths. Tissue hypoxia leads to a decrease in the proportion of oxygenated hemoglobin and myoglobin.^37^^,^^38^ Moreover, the proportion of oxygenated proteins measured in the ischemic region fluctuated between 0% and 19%. This phenomenon can be attributed to the interruption of blood flow to the myocardial apex in the experimental group due to coronary artery ligation, leading to persistent hypoxia in this region.^39^ In the myocardial area supplied by the ligated coronary artery, a portion of the myocardium experiences complete ischemia due to the absence of collateral circulation, resulting in the conversion of almost all hemoglobin and myoglobin into reduced forms. However, a small fraction of the myocardium may retain some metabolic capacity through minimal collateral circulation or diffusion of oxygen molecules from adjacent normal myocardium through intercellular spaces, thus preserving a portion of oxygenated proteins.^40^^,^^41^ In the control group, despite the absence of coronary artery ligation, some rabbits still exhibited a slight decrease in myocardial PA signals 3 h post-surgery, potentially due to chronic hypoxia caused by persistent slow blood loss after thoracotomy, leading to systemic hypoxia in the rabbits.^42^

There are several limitations in this study. Due to the limited thoracic size of rabbits, the probe designed for this study has a reduced contact area, which restricts the imaging field of view. As a result, the resulting photoacoustic images did not clearly reveal the microstructures of the myocardium. The primary objective of this study was to demonstrate the feasibility of using PAI for evaluating myocardial metabolic level, rather than pursuing high image resolution. Subsequent animal experiments on larger animals such as pigs will employ a larger semi-arc probe, which is expected to improve image quality and resolution. The sample size is insufficient for quantitative analysis of rabbit ${\mathrm{SmO}}_{2}$. Furthermore, the study did not involve a comparison with the widely accepted PET-CT due to the lack of small-animal PET-CT imaging equipment. In addition, due to the limitations of the probe and the size of the animal’s thoracic cavity, PAI was only able to capture ischemic regions distal to the ligation point, lacking information from non-ischemic areas for self-comparison. We will refine the animal models and upgrade the imaging system to enable more comprehensive investigation of myocardial characteristics using PAI.

## Data Availability

The datasets generated and analyzed during this study are available from the author upon reasonable request. Please contact: shiyw22@mails.tsinghua.edu.cn.

## Key Points

Using handheld photoacoustic imaging to evaluate rabbit myocardial metabolic status in an open-heart surgery model.
